# hMSH5 Regulates NHEJ and Averts Excessive Nucleotide Alterations at Repair Joints

**DOI:** 10.3390/genes13040673

**Published:** 2022-04-11

**Authors:** Aneesa T. Al-Soodani, Xiling Wu, Nicole C. Kelp, Alexander J. Brown, Steven A. Roberts, Chengtao Her

**Affiliations:** School of Molecular Biosciences, Washington State University, Pullman, WA 99164, USA; aneesa.als@gmail.com (A.T.A.-S.); wu_xiling@yahoo.com (X.W.); nicole.kelp@colostate.edu (N.C.K.); alexander.brown@wsu.edu (A.J.B.); steven.roberts2@wsu.edu (S.A.R.)

**Keywords:** MSH5, DSB repair, NHEJ, end resection, 53BP1, genome instability

## Abstract

Inappropriate repair of DNA double-strand breaks (DSBs) leads to genomic instability, cell death, or malignant transformation. Cells minimize these detrimental effects by selectively activating suitable DSB repair pathways in accordance with their underlying cellular context. Here, we report that hMSH5 down-regulates NHEJ and restricts the extent of DSB end processing before rejoining, thereby reducing “excessive” deletions and insertions at repair joints. RNAi-mediated knockdown of hMSH5 led to large nucleotide deletions and longer insertions at the repair joints, while at the same time reducing the average length of microhomology (MH) at repair joints. Conversely, hMSH5 overexpression reduced end-joining activity and increased RPA foci formation (i.e., more stable ssDNA at DSB ends). Furthermore, silencing of hMSH5 delayed 53BP1 chromatin spreading, leading to increased end resection at DSB ends.

## 1. Introduction

The formation and processing of physiologic DSBs is essential for the successful completion of meiosis and immunoglobulin gene rearrangements. On the other hand, however, DSBs are also generated pathologically by reactive oxygen species, arising amidst oxidative metabolism or exposure to genotoxic agents such as ionizing radiation (IR) and chemotherapeutic compounds [1,2]. It is estimated that, based on analyzing metaphase chromosomes, an average human cell can experience 10 DSBs per day [3]. Being the most deleterious DNA lesions in the genome, the biological impacts of DSBs can be extremely detrimental to cells if they are not repaired properly. Two-ended DSBs are predominantly repaired by NHEJ, whereas one-ended DSBs—often occurring during replication of DNA possessing single strand breaks—are preferably repaired by HR [4]. However, the repair of one-ended DSBs near telomeres by break-induced replication (BIR) could lead to genome rearrangement and cancer development [5]. DNA ends from the same DSBs are usually tracked and tethered to prevent inappropriate end-joining events that might lead to genome rearrangements such as deletions and/or chromosome translocations [6]. Our previous studies demonstrate that hMSH5 promotes DSB repair by HR [7,8]. The current study aimed to understand the role of hMSH5 in NHEJ-mediated DSB repair.

NHEJ represents a major end-joining activity, which is available throughout the cell cycle and especially important during the G1 phase [2]. The canonical (classical) NHEJ pathway (hereafter referred to as NHEJ) utilizes a collection of conserved factors including Ku70/Ku80, DNA-PKcs, and the ligase complex XRCC4-Ligase IV-XLF. The Ku70/Ku80 heterocomplex is among the first to bind the ends of DSBs to protect them from nucleolytic degradation and to recruit downstream NHEJ factors [9,10]. DSBs with compatible ends can be rejoined with little or no end processing, whereas, prior to ligation, incompatible ends have to be modified, leading to deletions, insertions, and sequence rearrangements at repair joints [11,12,13]. Besides the NHEJ pathway, recent evidence points to the existence of alternative end-joining (alt-NHEJ) activities [14].

On the other hand, HR-mediated DSB repair proceeds in a more orderly fashion and is normally constrained to the S and G2 phases of the cell cycle, during which homologous sequences are readily available [15,16]. Although HR can take place in several different forms and give rise to diverse outcomes, the initiation of HR is invariant, as it all requires the generation of single-stranded DNA ends by a two-tiered 5′-3′ end resection event [17]. The initial short end resection requires CtIP-promoted nuclease activities of the MRE11 complex [18,19]. DNA ends with short end resections are further processed by the BLM-DNA2-RPA-MRN or the EXO1-BLM-RPA-MRN nuclease complex to generate long resected ends [20,21]. This allows the subsequent formation of long, single-stranded DNA-Rad51 filaments to commence HR [22]. Studies in recent years have shown that end resection is governed by the interplay between 53BP1 and BRCA1. In particular, 53BP1, together with its effector Rif1, restricts the access of nucleases and therefore promotes NHEJ by limiting end resection [23,24]. On the contrary, BRCA1 promotes HR, at least partially, through antagonizing the interaction between 53BP1 and DSB-blemished chromatin [20]. Besides promoting NHEJ in G1, 53BP1 has also been suggested to foster Rad51-depenent HR while blocking Rad52-dependent single strand annealing in S/G2 phases of the cell cycle [25]. In the absence of extended end resection, NHEJ, but not HR, can also rejoin DNA ends possessing short ssDNA after proper modification. In addition, these breaks can also be processed and rejoined by activities that are not supported by the NHEJ pathway [26,27,28,29,30]. It is commonly believed that these non-canonical end-joining activities are frequently associated with more extensive sequence alterations at the DSB repair joints. For instance, it has been recently suggested that Polθ plays a role in promoting end-joining of resected DSBs through the highly mutagenic microhomology (MH)-mediated strand annealing mechanism [31,32,33,34].

In the present study, we investigated the regulation of NHEJ by the human MutS homologue MSH5 (hMSH5) [35]. MSH5 protein and its interacting partner MSH4 were initially identified and found to function in meiotic recombination in yeast and mice [36,37,38,39,40,41,42]. Our previous studies identified hMSH5 and found it promotes HR and facilitates the repair of cisplatin-induced DSBs [7,8]. In addition, hMSH5 promotes c-Abl activation and modulates c-Abl-p73-dependent cellular responses to IR-induced DNA damage, while it also plays a role in mitochondria DNA repair [43,44]. The action of hMSH5 in HR is closely connected with the DSB repair functions of hMRE11 and Rad51 [8,45]. The formation of cisplatin-induced hMSH5 foci requires Rad51, and the phosphorylation deficient hMSH5 mutant sensitizes cells to cisplatin, presumably through compromised Rad51 chromatin recruitment [7,8]. Furthermore, through interacting with FANCJ, hMSH5 promotes HR and ART-Chk1 signaling in response to anticancer agent Camptothecin [46]. Studies have also shown a connection between an hMSH5 variant and an increased use of microhomology at Ig switch joints in common variable immune deficiency (CVID) and IgA deficiency patients [47]. Here, we report that hMSH5 down-regulates NHEJ and protects DSB ends from extensive end processing. Conversely, hMSH5 deficiency leads to large nucleotide deletions as well as insertions at the repair junctions, presumably through delayed 53BP1 foci formation. Collectively, our current studies implicate a role for hMSH5 in the suppression of “mutagenic” NHEJ (i.e., excessive deletion and insertions at the repair joints), thereby abating DNA damage-induced genome alteration.

## 2. Materials and Methods

### 2.1. Plasmids and Expression Constructs

The shRNA plasmid, pmH1P-bsd/hMSH5 sh2 [44], was used to silence the expression of hMSH5 in all relevant experiments. The full-length and the N-terminally truncated hMSH5, hMSH5ΔN (lacking the first 115 amino acids), were expressed from pcDNA6(bsd)/flag-based expression constructs and detected by an anti-hMSH5 antibody created previously [44,48]. The RNAi-resistant version of hMSH5 expression construct was derived from pcDNA6(bsd)/flag-hMSH5 in which three silent mutations were introduced at the shRNA target (5′-TGGGCCTGAG**A**GA**C**GC**G**TG) on exon 13 between nucleotides 1031–1049 of the hMSH5 ORF. The NHEJ reporter locus #8-1 was cloned into the pPuro-Flag vector to generate the NHEJ reporter construct [49,50]. The I-*Sce*I endonuclease was expressed from either pCBA-(I-*Sce*I) or pCMV(I-*Sce*I)3xNLS plasmid [8,51,52]. Transient GFP protein expression was carried out by using pEGFPC1 (Clontech, Mountain View, CA, USA). The Cas9 encoding plasmid was obtained from Addgene. The sgRNA sequence targeting the NHEJ locus #8-1 was cloned into pmH1P to yield sgRNA/EJ vector.

### 2.2. Cell Culture, Transfection, and IR Irradiation

The Washington State University Institutional Review Board has approved the use of the human cell lines included in the current study. HEK293T and U2OS cell lines, as well as their derivatives, were all maintained in DMEM (GE Healthcare Life Sciences, Marlborough, MA, USA) supplemented with 5% fetal bovine serum (Atlanta Biologicals, Lawrenceville, GA, USA) and 5% newborn bovine serum (GE Healthcare Life Sciences) plus 1X antibiotic-antimycotic (Invitrogen, Grand Island, NY, USA). Cells were cultured in a 5% CO_2_ incubator at 37 °C. Cell lysates were routinely prepared in CelLytic m Mammalian Cell Lysis/Extraction Reagent (Sigma-Aldrich, St. Louis, MO, USA) containing 1X protease inhibitor cocktail (Thermo Fisher Scientific, Rockford, IL, USA). Transfections were carried out either by a standard calcium-phosphate procedure [53] or by the use of an Amaxa Nucleofector (Lonza Group Ltd., Allendale, NJ, USA). Stable transfectants were selected by 2.5 μg/mL puromycin or 10 μg/mL blasticidin (Invitrogen) for approximately one month, and single clones were expanded and validated by immunoblotting. IR irradiation was carried out at the Washington State University Nuclear Radiation Center with a cobalt-60 source at a dose rate of 4.45 Gy/min.

### 2.3. Hi-Throughput Sequence Analysis of DSB Repair Junctions

CRISPR was used to introduce a defined DSB in the NHEJ reporter cells transfected with either pcDNA6(bsd)/flag-hMSH5 or pmH1P-bsd/hMSH5 sh2 [44]. Genomic DNA was extracted five days after transfection. NHEJ junctions on NHEJ locus #8-1 were PCR amplified using unique barcoded primers. Sequencing of PCR samples was performed with Illumina MiSeq at the University of Idaho. The resulting fastq files were appropriately separated into sample specific files by barcodes. Prior to mapping to a reference sequence using Geneious 8 software, overlapping paired reads were merged and trimmed to begin at the sequence ‘ATAC’, 103-bp upstream of the Cas9 cleavage site, and to end at the sequence ‘TGCC’, 99-bp downstream of it. The resulting assembly was exported to a SAM format, enabling the categorization of each repair junction into three groups using custom Python scripts: junction with deletion, junction with insertion, and complex junctions containing both deletion and insertion. Detailed sequence information such as deletion length, insertion length, and MH usage at repair joints were also determined. Statistical analysis of the effects of hMSH5 on repair joints was carried out by the box-and-whisker plot and the Mann–Whitney U-test.

### 2.4. NHEJ Assay and Repair Junction Analysis

To perform the in vitro NHEJ analysis, cell extracts were prepared from one-liter suspension cultures, and the end-joining reactions were carried out at room temperature. The reaction was terminated by adding 2 μL of 0.5% SDS, 2 μL of 0.5 M EDTA, and 1 μL of 10 mg/mL protease (Sigma-Aldrich), followed by a 30 min incubation at 37 °C. NHEJ products were separated by agarose gel electrophoresis and were visualized and quantified after ethidium bromide staining [49,54]. To immunodeplete hMSH5 from cell extracts, 50 μL of 293T#8-1/hMSH5 cell extracts were incubated with 2 μL of either mouse pre-immune or anti-hMSH5 serum for 1 h at 4 °C. Then, 16 μL of 50% slurry of BSA-saturated Protein A/rProtein G-Agarose beads (Invitrogen) were added and incubated for another 1 h at 4 °C. The agarose beads were removed from the extracts by centrifugation at 750 g for 1 min at 4 °C. The supernatants were used in the DNA end-joining assay.

**Figure 1:**
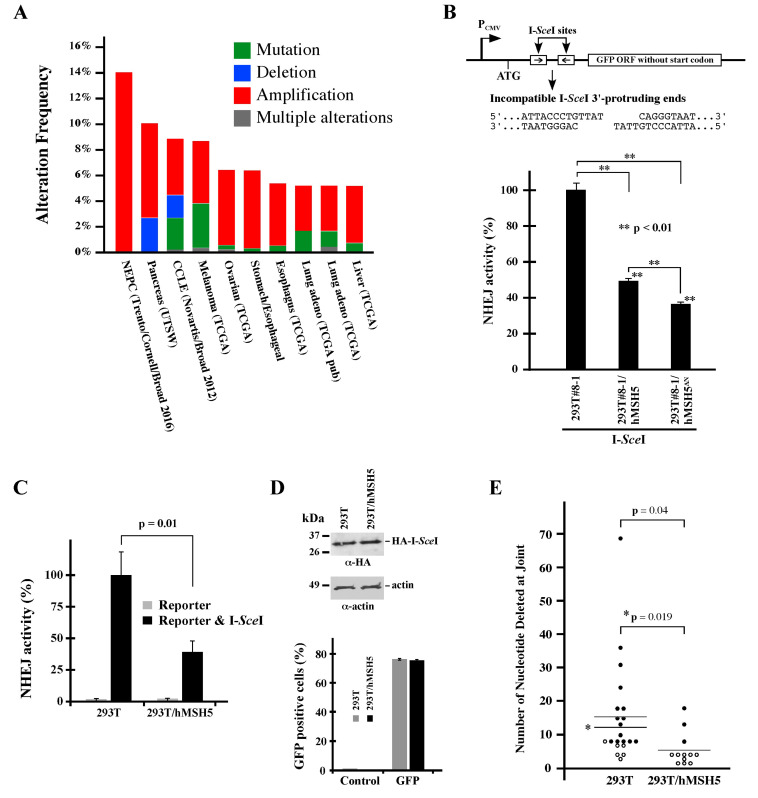
hMSH5 suppresses NHEJ-mediated DSB repair. (**A**) Analysis of the hMSH5 gene alteration in cancers. Data were retrieved from cBioPortal for Cancer Genomics (www.cbioportal.org). The stacked column graphs summarize 10 TCGA studies, of which each study has a sample size greater than 100, with at least 5% of the sample showing hMSH5 gene alterations. NEPC, neuroendocrine prostate cancer; CCLE, cancer cell line encyclopedia. (**B**) Schematic illustration of the NHEJ reporter locus in reporter cell line 293T/#8-1 [49]. NHEJ reporter analysis of the effect of hMSH5ΔN (hMSH5 aa116-834) (Tompkins et al., 2009). The cell lines used in this test were 293T/#8-1 derivatives stably expressing hMSH5 or hMSH5ΔN. (**C**) Analysis of the effect of hMSH5 on episomal NHEJ. 293T and 293T/hMSH5 cells were transiently transfected with either the NHEJ reporter construct alone or together with I-*Sce*I. (**D**) Levels of I-*Sce*I expression in 293T and 293T/hMSH5 cells determined by immunoblotting. The transfection efficiencies of 293T and 293T/hMSH5 cells (76% and 75%, respectively) were determined by transient transfection of pEGFP-C1, while untransfected cells were used as controls. (**E**) Sequence analysis of DSB repair junctions. The NHEJ reporter plasmid, together with I-*Sce*I construct, was transfected into 293T and 293T/hMSH5 cells. After inducing NHEJ-mediated end-joining at the reporter locus, repair joints were recovered by PCR amplification from total DNA. Cloned PCR products were sequenced. Sequencing data were analyzed by Tatsuki’s Dot Plot to reveal nucleotide deletions at the repair junctions. Solid circles signify repair joints without any I-*Sce*I 3′-protruding nucleotides (**B**, **top**), whereas open circles denote the inclusion of at least one of the 3′-protruding nucleotides at the repair junctions. Asterisks denote a similar statistical analysis in which the outlier (deletion of 69 nts) was omitted. Error bars represent standard deviations from the means of three replicates. Statistical significance was assessed by Student’s two-tailed *t*-test.

The in vivo NHEJ reporter analysis and the in vivo NHEJ assay [49] were used to analyze the effects of hMSH5. The in vivo NHEJ reporter contains two inverted I-*Sce*I recognition sites in between an ATG start codon and a linker sequence immediately 5′ to an ATG-less GFP coding sequence in such a way that the start codon is not in-frame with the GFP coding sequence (Figure 1B). However, NHEJ-mediated repair of I-*Sce*I-induced DSBs can position the ATG start codon in-frame with the GFP coding sequence, leading to GFP expression.

To analyze repair junction sequences, the NHEJ reporter and pCBA-(I-*Sce*I) plasmids were transiently transfected into 293T and 293T/hMSH5 cells. Four days later, total DNA was isolated from transfected cells by using a Blood and Cell Culture DNA Mini Kit (Qiagen, Valencia, CA, USA). Purified DNA was digested by I-*Sce*I (New England Biolabs, Ipswich, MA, USA) to eliminate uncut or rejoined I-*Sce*I sites. The repaired junctions were PCR-amplified (~500 bp) and cloned into the pDrive-cloning vector (Qiagen). Individual clones were expanded and DNAs were isolated using the Wizard Plus SV Minipreps DNA Purification System (Promega, Madison, WI, USA) and sequenced.

### 2.5. Pulsed-Field Gel Electrophoresis

Approximately 5 × 10^6^ 293T#8-1 and 293T#8-1/hMSH5 cells were treated with 20 Gy IR and harvested at indicated time intervals. Collected cells were then washed twice with ice-cold PBS and resuspended at 2 × 10^6^ cells/mL in PBS at 37 °C. Cells were then mixed with equal volume of 1.2% low melting agarose in PBS at 55 °C. The cell-agarose mixture was loaded as 100 μL aliquots into plug molds and left at 4 °C to solidify. Agarose plugs were treated with NDSK buffer (0.5 M EDTA, pH 8.0, 1% N-laurylsarcosine, 1 mg/mL Proteinase K) at 50 °C for 48 h. All plugs were stored in NDSK buffer at 4 °C. To perform PFGE, a 0.8% agarose gel was prepared in 0.5% TBE, and plugs were sequentially washed with TE, TE containing 100 μM PMSF, and TE. PFGE was carried out on a Bio-Rad CHEF DR III system at 2 V/cm gradient voltages, a 106° angle, and a 60–120 s switch time. The gel was run for 96 h at 14 °C and then stained with ethidium bromide for visualization and quantification. To quantify DNA fragmentation, all digital gel images were analyzed using the ImageJ software (U.S. National Institutes of Health, Bethesda, MD, USA). The background-corrected pixel densities of the samples at each time point following irradiation were compared to that of the non-irradiated controls to determine fold changes.

### 2.6. Immunofluorescence Microscopy

To analyze BRCA1, RPA, and 53BP1 foci formation, U2OS cells were grown on glass cover slips and either pre-extracted (for BRCA1 foci analysis) with extraction buffer (3 mM MgCl_2_, 0.3 M sucrose, 25 mM HEPES, 25 mM NaCl, 25 mM EDTA, and 0.5% Triton X-100) for 5 min at room temperature, and then fixed with 4% PFA for 15 min, or fixed directly with PFA without pre-extraction. Fixed cells were washed with PBS, permeabilized with 0.5% Triton X-100 for 20 min, and then blocked with 10% NBS in PBS for 30 min. Primary antibody incubations were for 1 h at room temperature, except for the RPA antibody, which was incubated at 37 °C. Antibodies and dilution ratios used in this study were: RPA2 (1:200) (Novus Biologicals, Littleton, CO, USA), BRCA1 D-9 (1:200) (Santa Cruz Biotechnology, Santa Cruz, CA, USA), and 53BP1 (1:500) (Novus Biologicals). Cells were washed 3 times in PBS prior to incubation with either Alexa 488 or 555-conjugated secondary antibodies (Life Technologies/Invitrogen, Grand Island, NY, USA) diluted in 10% NBS (1:500) for 1 h at room temperature. This was followed by 3 more washes in PBS, and then cells were mounted onto glass slides with Prolong Gold Antifade reagent with DAPI (Invitrogen/Life Technologies).

### 2.7. DNA End-Resection Analysis

Cells were grown in culture media containing 20 µM BrdU (Sigma-Aldrich) for 24 h and then subjected to the IF protocol with pre-extraction as described above. A BrdU antibody (1:200) (BD Pharmingen, San Jose, CA, USA) was used to detect BrdU foci (BrdU-incorporated ssDNA). To confirm that all cell preparations have equivalent levels of BrdU chromosomal incorporation, cells were fixed with PFA and permeabilized with Triton X-100, then treated with 2N HCl to denature DNA prior to BrdU immunostaining. Three examinations, each with 100 cells, were analyzed for every treatment condition and time point. Images were captured using a Leica Leitz DMRB microscope (Leica Microsystems, Wetzlar, Germany).

## 3. Results

### 3.1. Dysregulation of hMSH5 Is Common in Cancers

Previous studies have shown that hMSH5 is involved in DNA damage repair and particularly promotes HR [7,8,43,44,45,46]. As inappropriate execution of DSB repair increases the risk of genome instability and promotes cancer development [55], we first surveyed the TCGA database and found that the hMSH5 gene was frequently altered in cancers. There are presently 10 TCGA studies, each with a sample size over 100, showing hMSH5 alteration in at least 5% of the samples (cBioPortal for Cancer Genomics, www.cbioportal.org, accessed on 28 April 2017). Noticeably, the most common type of hMSH5 gene alteration, revealed by these relatively large-scale studies, is copy number increase (i.e., amplification) followed by mutation and deletion (Figure 1A). We therefore sought to determine the effects of hMSH5 deficiency and hMSH5 overexpression on DSB end-joining repair to establish a potential link between hMSH5 alteration and genomic instability.

### 3.2. hMSH5 Down-Regulates NHEJ Repair and Modulates DSB End Processing

To investigate the potential role of hMSH5 in NHEJ-mediated DSB repair, we analyzed the effects of hMSH5 on DNA end-joining activity using a chromosomal reporter locus stably integrated into the genome of 293T cells (the resulting reporter cell line is referred to as 293T#8-1). In this system, DSB-induced NHEJ activities can be monitored by the appearance of GFP-positive cells (Figure 1B, top) [49]. Using this reporter cell line, we found that stable hMSH5 overexpression reduced end-joining frequency by ~50% in I-*Sce*I-induced NHEJ repair (Figure 1B, bar graph). It is known that the hMSH5-interacting protein hMSH4 possesses an hMSH5-indepentent inhibitory activity on NHEJ (Chu et al., 2013). To confirm that the observed effect of hMSH5 on NHEJ was not related to hMSH4, a N-terminal truncated hMSH5 mutant (hMSH5ΔN), lacking the hMSH4-interacting domain [44,50,56], was examined. The results indicated that hMSH5ΔN could effectively suppress NHEJ repair (Figure 1B, bar graph), suggesting that the effect of hMSH5 on NHEJ is independent of hMSH4 as well.

Furthermore, this effect of hMSH5 on NHEJ could be recapitulated by transient transfection of the same NHEJ reporter and I-*Sce*I constructs into 293T and 293T/hMSH5 cells (Figure 1C). Expression control experiments confirmed a similar expression level for I-*Sce*I in 293T and 293T/hMSH5 cells, and the transfection efficiencies of these two cell lines were also similar as assessed by transient GFP expression (Figure 1D). These experiments indicate that the reporter locus can be used as either a chromosomal or an episomal NHEJ reporter. Next, we amplified repair joints from episomal NHEJ reporters, and the PCR products were then sequenced and analyzed. We found that the nucleotide deletions at the repair joints were significantly shorter in 293T/hMSH5 cells, raising the possibility that hMSH5 might possess the ability to restrict the extent of DSB end processing before rejoining (Figure 1E). It is worth noting that most repair joints that were recovered from 293T/hMSH5 cells contained at least one of the 3′-protruding nucleotides (Figure 1E, open circle). In addition, approximately 33% of joints that were recovered from 293T/hMSH5 cells and about 19% from 293T cells possessed MH, in which all MHs were composed of 1–3 nucleotides.

**Figure 2:**
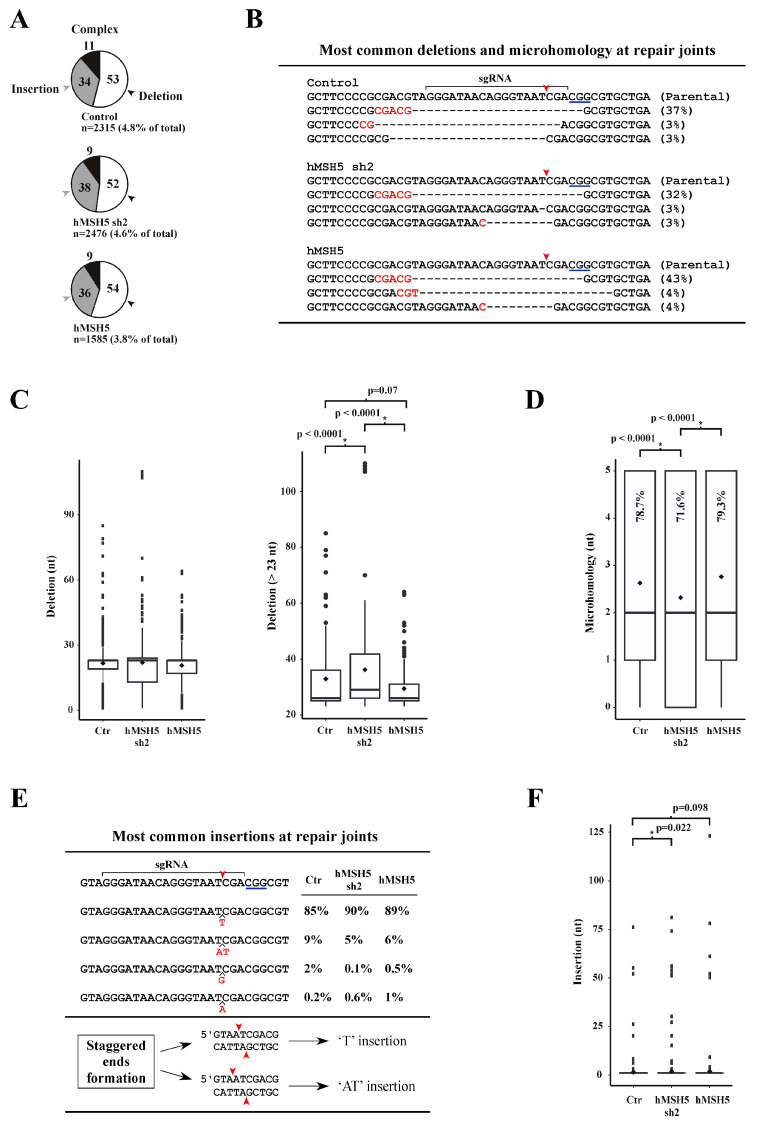
Hi-throughput sequence analysis of DSB repair junctions at the chromosomal NHEJ#8-1 locus. (**A**) Pie charts illustrate the divisions of sequence reads. Numbers inside the pie charts represent the percentages of each class of repair joints. (**B**) Sequence alignment of the most common deletions at repair joints. The occurrence of corresponding deletions is indicated by percentage. Red arrowheads mark the cleavage position by CRISPR/Cas9, and MHs are highlighted in red. (**C**) Box-and-whisker plot analysis of the length of deletion at repair joints. Height of the box is defined by the lower and upper quartiles, in which the thick line inside the box represents the median while the diamond represents the mean. Dots are values 1.5 times lesser or greater than the interquartile range. All sequences of 23-nt deletion but devoid of the 5-nt MH were included in the analyses. (**D**) Box-and-whisker plot analysis of MH length at repair joints. Percentages of junction sequences possessing MH are indicated for each category. (**E**) Compilation of the most common insertions at the repair joints. Inserted nucleotides are highlighted in red with their frequencies provided for each hMSH5 category. (**F**) Box-and-whisker plot analysis of insertion length at the repair joints. The Mann–Whitney U-test was used for statistical analysis, and *p* values were provided for those with statistical significance (in a few cases, non-significant *p* values were also provided for reference).

We next analyzed repair junction compositions from the chromosomal NHEJ#8-1 locus. The CRISPR/Cas9 system was adopted to introduce a DSB immediately after the second I-*Sce*I site (see Figure 1B, top). Repair joints were PCR amplified and sequenced by a next-generation approach aimed to recover all junction variations in cells expressing different levels of hMSH5 (i.e., parental, hMSH5 knockdown and overexpression). Approximately 3.8% to 4.8% of the total sequence reads were NHEJ joints that were composed of three categories: deletion, insertion, and complex (i.e., possessing both deletions and insertions) (Figure 2A). Overexpression of hMSH5 led to the lowest NHEJ events (3.8%) among the three hMSH5 conditions (Figure 2A). Based on the transfection efficiency shown in Figure 1D and the assumption that cells received both Cas9 and sgRNA would entail a DSB, we could deduce that approximately 90% of DSBs were rejoined without sequence alteration.

For repair joints with deletions, the most common type was a deletion of 23 nucleotides in which the repair joint possessed a 5-nt MH (the longest observed) shared between the first 5 nucleotides at the right-end and an internal region 19 nucleotides from the left-end of the DSB (Figure 2B). This repair junction—accounting for over 90% of repair joints with 23-nt deletion—represented 32% of repair joints in hMSH5-deficient cells, 37% in controls, and 43% in hMSH5 overexpressing cells (Figure 2B). Box-and-whisker plot analysis of sequences within the deletion category indicated that hMSH5 exerted an effect on deletion lengths at the repair joints. However, due to the over representation of the 23-nt deletion with 5-nt MH, statistical analysis did not indicate significant difference between hMSH5 conditions (Figure 2C, left). To remove the bias introduced by the 23-nt deletion with 5-nt MH, we further analyzed sequences of repair junctions with more than 23-nt deletions (Figure 2C, right). Box-and-whisker plot analysis of repair joints with more than 23-nt deletion showed that hMSH5 deficiency extended the length of nucleotide deletion, while its overexpression contracted it (Figure 2C, right). These observations support the hypothesis that hMSH5 exerts an effect on DSB rejoining through restricting DSB end processing. Repair joints in hMSH5-silenced cells tended to associate with shorter MHs and a decreased frequency of MH usage (Figure 2D).

Junctions with insertions were slightly over one-third of the total sequence reads; the major insertion was a ‘T’, and the next most common insertion was a dinucleotide ‘AT’—together, these two types accounted for ~95% of insertions. In contrast, ‘G’ or ‘A’ insertion events were significantly infrequent (Figure 2E). Evidently, hMSH5 deficiency led to longer insertions at the repair joints (Figure 2F). It is possible that the frequent insertion events were not a result of end processing and rejoining of the ends. Instead, the generation of ‘T’ and ‘AT’ insertions could be due to 5′-staggered cutting on the non-target strand by CRISPR/Cas9 prior to end processing and rejoining (Figure 2E). It is interesting to note that a recent study has demonstrated that the two DSB ends generated by CRISPR/Cas9 are processed differently, of which the end proximal to the protospacer adjacent motif has a lower chance to be altered [57]. Collectively, we found that hMSH5 plays a role in preventing excessive deletion at repair joints; and the increased NHEJ activity in hMSH5 deficient cells is expected to promote the joining of DNA ends with extensive processing.

### 3.3. hMSH5 Overexpression Decreases End-Joining and Delays IR-Induced DSB Repair

**Figure 3:**
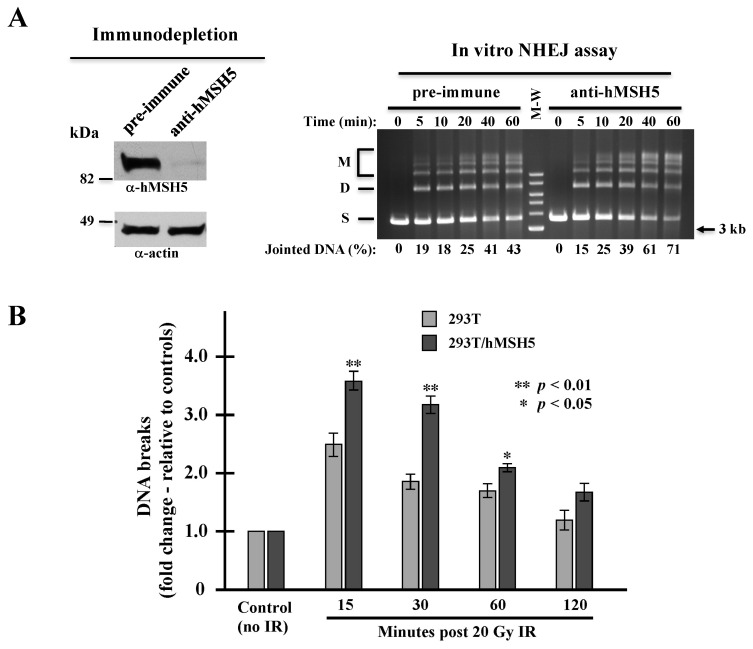
Effects of hMSH5 on NHEJ. (**A**) In vitro NHEJ analysis of NHEJ activities in hMSH5-depleted extracts in comparison to extracts depleted by pre-immune serum. Immunoblotting analysis of hMSH5 depletion from 293T/hMSH5 extracts is shown on the left. End-joining reactions were carried out by incubating cell extracts with *Sal*I-digested plasmid DNA as described previously [49]. ‘S’ represents linear DNA substrate, ‘D’ for joint dimer, and ‘M’ signifies higher order joint products. Relative NHEJ activities (%) were percentages of total substrate loss determined by quantification at each time point (right panel). (**B**) Assessing the effect of hMSH5 on IR-induced DSB repair by PFGE. The detection of DNA breaks in 293T and 293T/hMSH5 cells by PFGE analysis was performed at indicated times following IR exposure. Fold change in DNA fragmentation was determined by using an untreated sample as a reference. Error bars represent standard deviations from the means of three replicates. Statistical significance was determined by Student’s two-tailed *t*-test.

The above observations raised a possibility that hMSH5 might limit end-joining by enhancing MH usage, thereby delaying rejoining of DNA ends without MHs. We therefore carried out an in vitro NHEJ assay to analyze the effect of hMSH5 on NHEJ repair of DSBs with compatible ends. We found that 293T/hMSH5 extracts exhibited time-dependent NHEJ activities towards a linearized DNA substrate—generating both end-joined dimers and multimers (Figure 3A, right). By this assay, we found hMSH5 immunodepletion increased the end-joining activity (Figure 3A). This indicates that hMSH5 can also inhibit the joining of compatible DSB ends independent of MH status. This result suggests that the NHEJ suppressive activity of hMSH5 is not due to its stimulatory effect on MH usage.

Next, we investigated whether hMSH5 exerted a similar effect on NHEJ repair of biologically relevant DSBs. IR is the most common cause of pathological and therapeutic DSBs in which their repair is NHEJ-dependent [2]. It is known that the repair of IR-induced DSBs follows a biphasic mode—the fast repair component (i.e., NHEJ) is able to repair approximately 80% of the DNA breaks within the first 2 h, while the slow component continues to repair the remaining breaks for up to 24 h [58,59,60]. The physiological implications of hMSH5 on NHEJ were assessed by pulsed-field gel electrophoresis (PFGE) analysis of 293T and 293T/hMSH5 cells at various time points after IR treatment. In comparison to 293T cells, hMSH5 overexpression increased the levels of chromosome fragmentation at all time points examined, but to a much greater extent during the first half hour following IR exposure (Figure 3B). These observations suggest that hMSH5 exerts a negative impact on NHEJ-mediated DSB repair. It is conceivable that elevated NHEJ activity in hMSH5-depleted cells may facilitate the fixation of sequence alterations at the repair junctions.

### 3.4. hMSH5 Depletion Heightens the Processing of IR-Induced DNA Breaks

**Figure 4:**
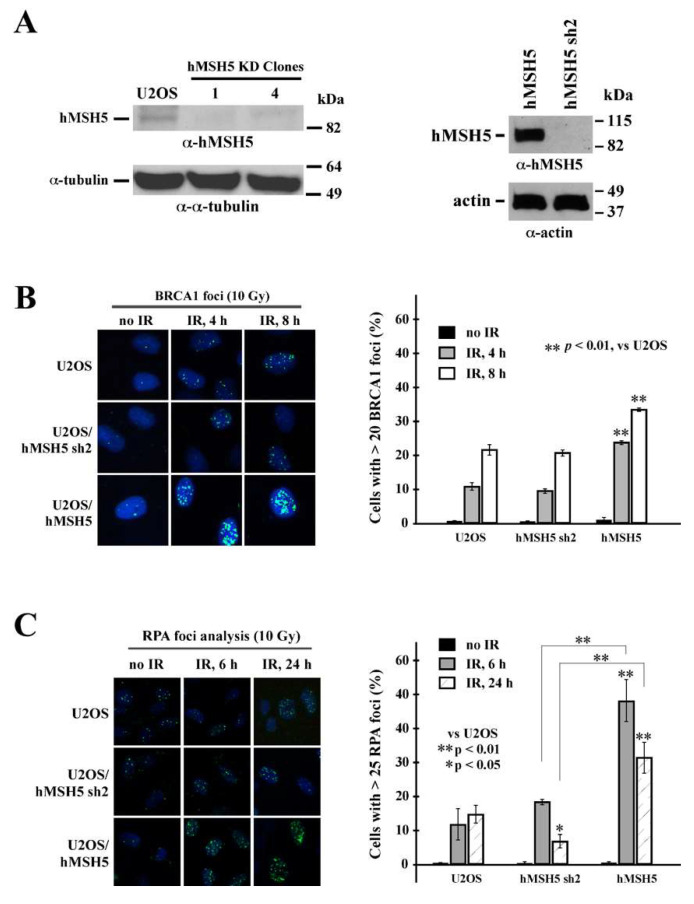
Analysis of the effect of hMSH5 on IR-induced BRCA1 and RPA foci formation. (**A**) Immunoblotting analysis of RNAi-mediated hMSH5 silencing (by hMSH5 sh2) in two representative stable clones, of which clone#1 was used for all subsequent studies. Immunoblotting of U2OS/hMSH5 stable transfectant is shown on the right. (**B**) BRCA1 foci formation at 4 or 8 h after 10 Gy IR treatment in U2OS, U2OS/hMSH5 sh2, and U2OS/hMSH5 cells. A panel of representative images of BRCA1 foci is provided on the left. Three separate foci counts, each with 100 cells, were analyzed for every treatment condition and time point. The mean percentages of cells possessing 20 or more BRCA1 foci/nucleus were graphically displayed (on the right) together with their corresponding standard deviations (error bars). (**C**) RPA foci formation at 6 and 24 h post 10 Gy IR in U2OS, U2OS/hMSH5 sh2, and U2OS/hMSH5 cells. Representative images of RPA foci for all cell lines are shown on the left. Bar graphs are employed to show the percentage of RPA foci formation (>25 foci). Asterisks are used to indicate statistically significant differences between categories. Error bars are standard deviations of the means that were determined from three 100-cell replicates. Statistical significance was assessed by Student’s two-tailed *t*-test.

To determine the effects of hMSH5 on the processing of DSB ends before rejoining, we first examined its effects on DSB-induced recruitment of pro-HR factor BRCA1 and ssDNA binding protein RPA. In parental U2OS and hMSH5-silenced or -overexpressed cells (Figure 4A), we found that hMSH5 depletion exerted no effect on IR-induced BRCA1 foci formation (Figure 4B). In contrast, cells overexpressing hMSH5 exhibited moderately high levels of BRCA1 foci in response to IR treatment (Figure 4B). This is likely due to the HR-promoting property of hMSH5 [7,8]. BRCA1 is known to play a critical role in directing DSB repair toward HR by antagonizing the action of 53BP1—a key NHEJ determining factor that negatively regulates end resection [20,23,24]. The results of RPA foci analysis showed that hMSH5 depletion significantly decreased the levels of RPA foci (ssDNA) formation at 24 h after IR treatment (Figure 4C). The timing of this is within the slow phase of DSB repair [58,59,60]. In response to IR, hMSH5 overexpression in cells increased the levels of RPA foci formation (Figure 4C). It is possible that hMSH5 depletion might lessen an appropriate protection for the ssDNA at the break ends, thereby leading to larger deletions at the repair joints.

**Figure 5:**
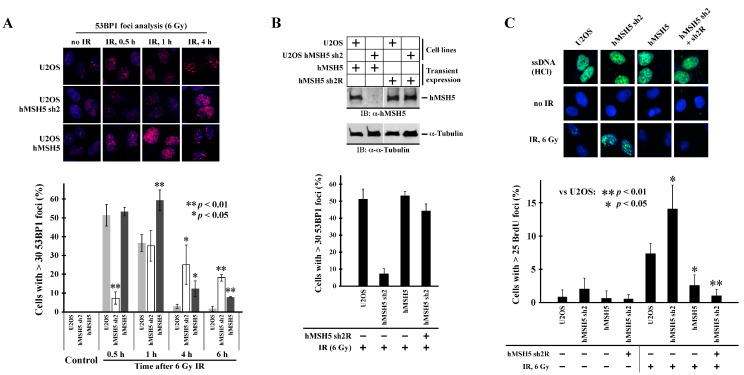
Effects of hMSH5 on IR-induced 53BP1 foci formation and DSB end resection. (**A**) Examination of IR-induced 53BP1 foci formation in U2OS, U2OS/hMSH5 sh2, and U2OS/hMSH5 cells at 0.5, 1, 4, and 6 h after irradiation with 6 Gy IR. A representative image panel of 53BP1 foci for each cell line is provided. Thirty foci per cell were used as the cutoff to analyze the effect of hMSH5 on IR-induced 53BP1 foci formation. Bar graphs are used for data presentation, and asterisks are used to designate significant differences among all three cutoff categories. Error bars are standard deviations determined from three counts, each with 100 cells. (**B**) Rescuing of IR-induced 53BP1 foci formation by RNAi-resistant hMSH5 (sh2R) at 30 min post IR treatment. Upper panel shows immunoblotting analysis of RNAi-resistant hMSH5 expression in U2OS/hMSH5 sh2 cells. (**C**) Examination of ssDNA formation in U2OS, U2OS/hMSH5 sh2, U2OS/hMSH5, and hMSH5-complemented U2OS/hMSH5 sh2 cells. A panel of representative BrdU immunostaining images is included to show equivalent levels of BrdU incorporation in all cell lines (**top**) and the IR-induced BrdU foci formation (ssDNA) at 30 min after exposure to 6 Gy IR (**middle** and **bottom**). Percentages of cells with >25 BrdU foci, determined by three counts, each with 100 cells, are graphically displayed with error bars representing standard deviations. Statistical significance was determined by Student’s two-tailed *t*-test.

One factor that controls ssDNA formation at DSBs is 53BP1. We therefore analyzed the effects of hMSH5 on IR-induced 53BP1 foci formation. As shown in Figure 5A, hMSH5 depletion significantly delayed IR-induced 53BP1 foci formation (>30 foci per cell) within the first half hour after IR treatment. The formation of 53BP1 foci was enhanced at 1 h post IR; in both hMSH5-deficient and -overexpressed cells, the disappearance of 53BP1 foci was significantly delayed within 4–6 h after IR exposure (Figure 5A), likely owing to the accumulation of unresolved DNA breaks. The initial delay of 53BP1 foci formation in hMSH5-deficient cells could render the ends of DSBs susceptible to resection, resulting in subsequent nucleotide deletions at the repair joints. To validate that the delay of 53BP1 foci formation was indeed caused by hMSH5 depletion, we performed a rescue experiment in which the RNAi-resistant hMSH5 expression construct hMSH5 sh2R was used to express hMSH5 in hMSH5-deficient U2OS cells (Figure 5B, top). Observably, the restoration of hMSH5 expression led to a near complete recovery of 53BP1 foci formation at 30 min after IR (Figure 5B). Finally, we examined whether the delay of 53BP1 foci formation enhanced end resection of IR-induced DSBs. To this end, we took advantage of the anti-BrdU antibody that only recognizes BrdU embedded in ssDNA but not in dsDNA [61]. In this approach, the accumulation of resected DSB ends was monitored by BrdU foci formation at 30 min after 6 Gy IR. To show equal chromosomal incorporation of BrdU in cells expressing different levels of hMSH5, total dsDNA was denatured by HCl to produce ssDNA. The levels of total chromosomal BrdU were then detected by anti-BrdU staining (Figure 5C, top row). Therefore, in the absence of HCl, ssDNA at the ends of DSBs can be easily detected by anti-BrdU antibody. Evidently, hMSH5 depletion significantly increased IR-induced BrdU foci formation, indicative of ssDNA formation (Figure 5C). Furthermore, expression of an RNAi-resistant hMSH5 (sh2R) in hMSH5-deficient cells could completely abolish the formation of BrdU foci (Figure 5C). These results indicated that the delay in 53BP1 foci formation promoted end resection at IR-induced DSBs. In short, we found that hMSH5 prevented excessive processing of break ends by facilitating IR-induced 53BP1 foci formation, and hMSH5 maintained RPA foci during the slow phase of end-joining repair.

## 4. Discussion

Cellular context-dependent execution of suitable DSB repair pathway is essential for preventing erroneous DNA repair that, if unleashed, can result in deleterious mutations, deletions, and genome rearrangements. In this study, we aimed to investigate the effects of hMSH5 on the rejoining of DSB ends, and we found that hMSH5 affects the accuracy of end-joining in NHEJ-mediated DSB repair. Specifically, hMSH5 depletion exaggerates IR-induced ssDNA formation through delaying 53BP1 foci formation. As 53BP1 plays a key role to block end resection [20,23,24], its delay is expected to aid ssDNA formation. In fact, the lack of 53BP1 at DSBs would create a window of opportunity for the hMRE11-CtIP complex to act on the ends of DNA breaks leading to short end resections. However, the generation of ssDNA by short end resection alone is not sufficient to engage HR, and eventually ends with short ssDNA are primarily repaired by NHEJ [62,63,64]. However, NHEJ-mediated rejoining of DSBs with resected ends requires ssDNA processing and that can increase deletions and/or rearrangements at the repair joints.

The hMSH5-interacting partner hMSH4 was previously shown to display a strong inhibitory effect on NHEJ [49]. Here, we found that the effect of hMSH5 on NHEJ was independent of its interaction with hMSH4 (Figure 1B). This raises the possibility that hMSH5 and hMSH4 play redundant roles in the negative regulation of NHEJ. Besides down-regulation of NHEJ-mediated repair of DSBs with either incompatible or compatible ends, hMSH5 also prevents excessive processing of DNA ends before rejoining. The effect of hMSH5 on reducing nucleotide deletions at NHEJ joints is likely a reflection of 53BP1 in harnessing initial nuclease-mediated end resection at DSBs. This function of hMSH5 is particularly pertinent to the repair of IR-induced DSBs that are inevitably devoid of blunt ends possessing 3′-hydroxyl and 5′-phosphate groups for direct ligation. In fact, IR-induced DSBs are composed of complex, often clustered, lesions and overhangs [2]. Successful rejoining of these DBSs requires end processing, in which hMSH5 deficiency is expected to result in excessive deletions at the joints. In addition, we found that hMSH5 overexpression decreased the overall NHEJ activities, presumably by shunting end-joining reactions to those relying on MHs, thereby hindering direct end rejoining. One previously postulated role of hMSH5 in HR-based DSB repair is to facilitate homology searching [8,45,46]. We also found that hMSH5 overexpression led to an increase in RPA and BRCA1 foci formation (i.e., enhanced end resection) between 4 to 8 h after IR exposure (Figure 4) which coincided with a delay in the disappearance of IR-induced 53BP1 foci (Figure 5A), likely resulting from unresolved DNA breaks. Together, these observations suggest that deviations from the physiological hMSH5 expression level could compromise timely DSB repair.

Although how hMSH5 facilities IR-induced 53BP1 foci formation remains to be delineated, it is possible that hMSH5 plays a role in the early stage of 53BP1 chromatin recruitment, and hMSH5 deficiency is expected to increase end resection. Rejoining of DSBs with extensively resected ends will likely contribute to genomic instability that can promote malignant transformation. Genome alteration, such as deletion, tends to accumulate in NHEJ-dysregulated cells; these cells, potentially oncogenic, can tolerate extensive DNA alteration and survive if p53 is also defective [65,66,67]. Our findings suggest that hMSH5 may play a dual role in cells suffering IR-induced genome alteration. High doses of IR (>2 Gy) are known to cause hMSH5 induction, and the elevated levels of hMSH5 promote c-Abl-dependent IR-induced apoptosis [44]. Thus, the net effect of hMSH5 is to reduce genomic instability—first by minimizing sequence alterations at the repair joints, and second by delaying NHEJ with concomitant heightening IR-induced apoptosis [44]. Furthermore, c-Abl kinase is known to negatively regulate the slow phase of NHEJ repair of IR-induced DSBs [68]. Therefore, hMSH5-dependent c-Abl activation also plays a role in minimizing DNA repair-induced genome alteration.

Interestingly, we found that silencing of hMSH5 disrupted the maintenance of RPA foci at 24 h after IR exposure (Figure 4C). The timing of this RPA foci reduction coincides well with the slow phase of DSB repair [58,59,60]. RPA binding to ssDNA is known to antagonize MH-mediated DSB repair [69]. Thus, it is plausible that hMSH5 depletion increases genome instability through either deletion of ssDNA at DSB ends (fast phase) or promotion of MH-mediated DSB repair (slow phase). Furthermore, increase in RPA foci, indicative of ssDNA formation, at 6 h after IR exposure was observed in hMSH5-overexpressing cells (Figure 4C). This is consistent with the increase in ssDNA formation at the early stage of the slow phase of DSB repair that can also favor the use of MH-mediated end-joining. Together, these results indicate that either up- or down-regulation of hMSH5 can render cells prone to DSB-induced genome alterations, supporting a role for hMSH5 dysregulation in cancer (e.g., TCGA database survey) (Figure 1A).

In addition to pathological DSB repair, our findings have implications for physiologic DSBs as well. It is known that hMSH5 functions in meiotic recombination and forms chromosome foci in fetal oocytes. During late zygonema, at the peak level, approximately 150 hMSH5 foci per nucleus can be readily detected [70]. Besides promoting HR during meiotic recombination, foci-associated hMSH5 can also function to prevent unwanted NHEJ events in meiosis. Furthermore, our current study suggests that hMSH5 deficiency reduces MH usage in end-joining repair. Long MH at the CSR joints has been previously associated with a disease-linked hMSH5 variant [47]. The reconciliation of these observations will settle on whether the disease-linked hMSH5 variant could act as a gain-of-function mutant. Finally, modulation of factors controlling NHEJ can be expected to have an impact on the effectiveness of DNA-damaging anticancer drugs. One reason that cancer cells survive genotoxic treatment is they can aberrantly regulate DSB repair and DNA damage response. Understanding of the precise molecular events underpinning the role of hMSH5 in harnessing NHEJ will provide an opportunity to manipulate DSB repair in cancer cells, thereby enhancing the therapeutic efficacy of DSB-inducing anticancer treatments.

## Data Availability

The data supporting hMSH5 alteration in cancers were retrieved from the TCGA database (cBioPortal for Cancer Genomics, www.cbioportal.org, accessed on 28 April 2017).
